# The effect of salidroside in promoting endogenous neural regeneration after cerebral ischemia/reperfusion involves notch signaling pathway and neurotrophic factors

**DOI:** 10.1186/s12906-024-04597-w

**Published:** 2024-08-01

**Authors:** Jiabing Zheng, Jizhou Zhang, Jing Han, Zhichang Zhao, Kan Lin

**Affiliations:** 1 Fujian Medical Universtity Union Hospital, Fuzhou, Fujian Province People’s Republic of China; 2 Institute of Materia Medica, Fujian Academy of Chinese Medical Sciences, Fuzhou, Fujian Province People’s Republic of China

**Keywords:** Salidroside, Cerebral ischemia/reperfusion, Ischemic stroke, Neural regeneration, Notch signaling pathway, BDNF

## Abstract

**Background:**

Salidroside is the major bioactive and pharmacological active substance in *Rhodiola rosea* L. It has been reported to have neuroprotective effects on cerebral ischemia/reperfusion (I/R). However, whether salidroside can enhance neural regeneration after cerebral I/R is still unknown. This study investigated the effects of salidroside on the endogenous neural regeneration after cerebral I/R and the related mechanism.

**Methods:**

Focal cerebral I/R was induced in rats by transient middle cerebral artery occlusion/reperfusion (MCAO/R). The rats were intraperitoneally treated salidroside once daily for 7 consecutive days. Neurobehavioral assessments were performed at 3 days and 7 days after the injury. TTC staining was performed to assess cerebral infarct volume. To evaluate the survival of neurons, immunohistochemical staining of Neuronal Nuclei (NeuN) in the ischemic hemisphere were conducted. Also, immunofluorescence double or triple staining of the biomarkers of proliferating neural progenitor cells in Subventricular Zone (SVZ) and striatum of the ischemia hemisphere were performed to investigate the neurogenesis. Furthermore, reverse transcription-polymerase chain reaction (RT-PCR) and enzyme-linked immunosorbent assay (ELISA) were used to detect the expression of neurotrophic factors (NTFs) brain-derived neurotrophic factor (BDNF) and nerve growth factor (NGF). Expression of Notch1 and its target molecular Hes1 were also analyzed by western-blotting and RT-PCR.

**Results:**

Salidroside treatment ameliorated I/R induced neurobehavioral impairment, and reduced infarct volume. Salidroside also restored NeuN positive cells loss after I/R injury. Cerebral I/R injury significantly increased the expression of 5-Bromo-2’-Deoxyuridine (BrdU) and doublecotin (DCX), elevated the number of BrdU/Nestin/DCX triple-labeled cells in SVZ, and BrdU/Nestin/glial fibrillary acidic protein (GFAP) triple-labeled cells in striatum. Salidroside treatment further promoted the proliferation of BrdU/DCX labeled neuroblasts and BrdU/Nestin/GFAP labeled reactive astrocytes. Furthermore, salidroside elevated the mRNA expression and protein concentration of BDNF and NGF in ischemia periphery area, as well. Mechanistically, salidroside elevated Notch1/Hes1 mRNA expression in SVZ. The protein levels of them were also increased after salidroside administration.

**Conclusions:**

Salidroside enhances the endogenous neural regeneration after cerebral I/R. The mechanism of the effect may involve the regulation of BDNF/NGF and Notch signaling pathway.

**Supplementary Information:**

The online version contains supplementary material available at 10.1186/s12906-024-04597-w.

## Introduction

Stroke is a major cause of mortality and morbidity in industrialized countries [1], with ischemic stroke accounting for almost 85% of all stroke cases [2]. The extremely high disability and mortality of the disease imposes an extremely heavy burden on families and society. Tissue plasminogen activator (t-PA) is currently the only therapeutic drug approved by US Food and Drug Administration (FDA) for ischemic stroke treatment. However, its treatment time window is only 3–4.5 h after onset [3], which is so narrow that a large number of the patients could not be rescued in the first place. Reducing infarct volume and promoting the generation of new neurons in the damaged brain are crucial approaches in stroke treatment. Yet effective drugs have not been developed. Increasing evidences indicate the existence of neural stem cells in specific brain regions such as the subventricular zone (SVZ) and dentate gyrus of the hippocampus, which are capable of self-renewal and multipotency [4]. Stimuli such as ischemia/hypoxia are reported to enhance neurogenesis in these regions. Promoting endogenous neural regeneration has emerged as a crucial therapeutic strategy for stroke [5].

Salidroside is the main active ingredient extracted from the roots and rhizomes of *Rhodiola rosea* L. As a glycoside2-(4-hydroxyphenyl) ethyl-β-dglucopyranoside (molecular formula: C14H20O7; molecular weight: 300.31; CAS registry number: 10338-51-9; PubChem CiD:159,278), salidroside is also found in other species such as *Fructus Ligustri Lucidi* [6], *Osmanthus fragrans* Lour [7]. and *Loranthus micranthus* Linn. [8], et al. In recent years, salidroside has received more and more attention because of its extensive pharmacological effects in numerous organ systems [9–12]. Especially, in vitro or in vivo experiments confirmed that salidroside has a promising therapeutic effect on disorders of nervous system. Recently, many researchers reported the effects of salidroside on cerebral ischemic injury [13]. Salidroside was found to be highly neuroprotective against ischemia/reperfusion (I/R) injury with a wide therapeutic time window [14, 15], and it exerts its neuroprotective effects on cerebral ischemia mainly through anti-oxidation and anti-inflammatory effects [16–19]. In our previous study, we have also reported that the Nrf2 pathway is involved in the anti-oxidant action of salidroside on cerebral ischemic injury [20]. And we also found that the neuroprotective effect of salidroside is associated with the modulation of dopaminergic system [21].

Notably, evidences indicated that salidroside may promote neurogenesis in rat model Alzheimer’s disease [22]. It induces the differentiation of mesenchymal stem cells (MSCs) into dopaminergic neurons [23]. Furthermore, salidroside was found to protect rat neural stem cells against hypoxia-induced injury [24]. These facts suggest that salidroside may have neurogenesis promotion effects. However, up to now, the effects of salidroside on endogenous neuroregeneration after cerebral ischemia have not been reported. Brain-derived neurotrophic factor (BDNF) and nerve growth factor (NGF) are members of the neurotrophic factor family, which are important regulators of neuroregeneration after cerebral ischemia. Salidroside was reported to up-regulate BDNF gene expression in a rat model of depression [25]. In MSCs, BDNF and NGF mRNA expression and protein secretion were increased after salidroside administration to promote the differentiation of MSCs into dopamine neurons [23]. Membrane protein Notch is one of the most important signaling molecules involved in cell proliferation, differentiation and development fate switching [26]. Upregulation of Notch signaling promotes neurogenesis after cerebral ischemia [27]. However, whether salidroside has an effect on endogenous neuroregeneration by influencing neurotrophic factors and Notch signaling pathway needs to be explored.

In the present study, we investigate the effect of salidroside on the neurological rehabilitation and endogenous neural regeneration in a rat model of focal cerebral I/R and explore its influence on neurotrophic factors and Notch signaling pathway. Since Notch1 and its downstream transcriptional target Hes1 mainly expressed in SVZ [28], we choose SVZ region to detect Notch signaling molecules to better elucidate the role of the Notch signaling pathway in promoting neural progenitor cell proliferation by salidroside.

## Materials and methods

### Animals

Male Sprague–Dawley rats (8-10-week-old), weighing 280 to 350 g, were purchased from SLAC Laboratory Animal Co. Ltd., Shanghai, China (SCXK (Hu)2019-0002). The animals were caged under 12-hour light/dark cycle with free access to standard diet and water. The study protocol was in accordance with the international laws on animal experimentation and was approved by the Committee of Ethics of Fujian Academy of Chinese Medical Science, China. (approval No. FJATCM-IAEC2021008)

### Transient middle cerebral artery occlusion (MCAO) model

The rats were anesthetized with 2–3% isoflurane at first, and then maintained anesthesia with 1–2% isoflurane using a small animal anesthesia machine (R580, RWD life Science Inc. Shenzhen, China). Focal cerebral I/R surgery was carried out based on the method used in our previous study [29]. The right common carotid artery (CCA), the external carotid artery (ECA) and the internal carotid artery (ICA) were exposed and isolated. A nylon thread with a silicon-coated tip (0.38 mm diameter for rats of 280–350 g) (Guangzhou Jialing Biotechnology Co., Ltd., Guangzhou, China) was inserted into the ICA to approximately 17–18 mm distal to the carotid bifurcation and occluded the middle cerebral artery (MCA). After 120 min, the suture was removed from the ICA to allow MCA reperfusion. During the surgical procedure, the body temperature was maintained between 36.5 and 37.0 °C. The rats in the sham operated group underwent all surgical procedures, but no suture was made.

In order to confirm the success of the MCAO model, neurological behavior was evaluated after reperfusion immediately by the 5-point scoring system as below: 0, no deficits; 1, difficulty in fully extending the contralateral forelimb; 2, difficulty in walking in straight line; 3, circling to the contralateral side; and 4, leaning to the contralateral side. Rats which scored below 3 were included in the following tests and were grouping according to their behavior scores to ensure consistent levels of neural damage at the beginning of the experiments. Totally 92 rats were selected in this study. All animals in the sham-operated group survived, and the overall mortality rate for MCAO surgery was 14.6% (12/82). All the animal grouping and their surgical mortality rate were listed in the Supplementary file (Supplementary Table 1).

### Experimental design and 5-bromo-2’-deoxyuridine (BrdU) injection

67 rats were divided into 5 groups as follows: the sham operation group (Sham, *n* = 17), the vehicle group (IscVeh, *n* = 17), and 3 salidroside treatment groups (IscSal20, IscSal40 and IscSal80, *n* = 8, 17 and 8 respectively). They were sacrificed at day 7 following the cerebral I/R for further analysis. Another 25 rats were divided into 3 groups as follows: sham operation group (Sham, *n* = 5), the vehicle group (IscVeh, *n* = 10), and salidroside treatment groups (IscSal40, *n* = 10). They were sacrificed at day 3 after the surgery to prepare brain slices for TTC, immunofluorescence and immunohistochemical staining. Salidroside (purity > 99%, Guangrun Biotech Co. Nanjing, China) was dissolved in 0.9% NaCl before use. The rats in IscSal20, IscSal40, IscSal80 groups were intraperitoneal administrated 20, 40, 80 mg/kg salidroside, once daily, started immediately after the cerebral I/R and continued through the indicated time period. Meanwhile the rats in the vehicle and sham group were administrated saline.

**Fig. 1:**
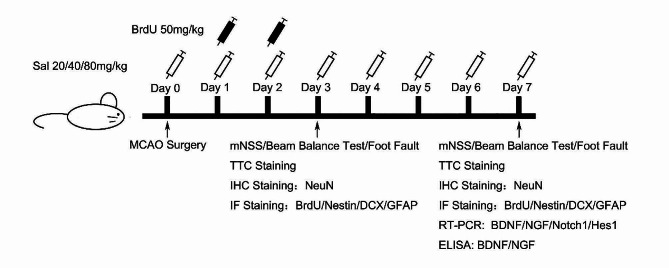
Schematic diagram of the experimental protocols

For the rats used to detect the neural regeneration, BrdU (Sigma, 50 mg/kg) was injected intraperitoneally at day1 and day2 after I/R for labelling proliferating cells. All the experimental schemes were shown in Fig. 1.

### Neurobehavioral assessment

To assess the effects of salidroside on the neurobehavioral function recovery, the modified neurological severity score (mNSS), foot fault test and beam balance test were performed at 3 and 7 days following the cerebral I/R by investigators who were blinded to the experimental groups.

### mNSS

The mNSS is a composite of motor, sensory, reflex, and balance assessment. The neurobehavioral deficits were scored using a modified scoring system based on [30]. The scores graded on a scale of 0 to 14 (normal score 0; maximal deficit score 14). In the neurological severity scores of injury, 1 point is awarded for the inability to perform the task or the lack of a tested reflex; thus, the higher score indicates a more severe injury.

### Beam balance test

Rats were put on an elevated balance beam (1.5 cm wide and 80 cm long; 15 cm high above a soft platform). The behavior performance was scored using a scoring system introduced by Chen et al. [30]: 0, balances with steady posture; 1, grasps side of beam; 2, hugs the beam and one limb falls down from the beam; 3, hugs the beam and two limbs fall down from the beam, or spins on beam (> 60s); 4, attempts to balance on the beam but falls off (> 40s); 5, attempts to balance on the beam but falls off (> 20s); 6, falls off: no attempt to balance or hang on to the beam (< 20s). A higher score means more severe impairment.

### Foot fault test

The foot fault test evaluated motor coordination ability of forelimb. The rats were placed on a 45 × 30 cm^2^ metal square grid, with each grid cell 2.5 × 2.5 cm^2^ and 8 cm high above the ground [31]. Under physiological conditions, rats preferred to place their paws at the intersection of the grid lines when traveling through the grid. After MCAO surgery, the rats could not place their paws accurately, and the forelimbs fall into the grid cells. Each time the paw falls down or slips through the grid lines, a foot fault is recorded. For each rat, the total number of steps on the grid (movements of each forelimb), as well as the whole number of foot faults in a 3-minute period was recorded. The foot fault score was represented as the percentage of foot faults over the total number of steps.

### 2,3,5-triphenyltetrazolium chloride staining

2,3,5-triphenyltetrazolium chloride(TTC)staining was used to measure the infarct volume. Rats were sacrificed under deep anesthesia with isoflurane. The brains were collected and washed with saline for 5 min. Then the brain was transferred into a brain mold and sliced into 5 evenly thick sections with a 2 mm interval. The sections were incubated in 1.5% 2,3,5-triphenyltetrazolium chloride at 37℃ for 30 min. During the period, the slices were flipped every 10 min and washed with ddH2O. After fixation in 4% paraformaldehyde overnight, images of the sections were captured using a digital camera. The infarct area (represented by unstained tissue) was measured using Image J 1.51 K software. The calculation of infarct volume was performed by researchers blinded to the experimental grouping. Infarct areas in all sections were combined to obtain a whole infarct area, which was then multiplied by the thickness of brain sections to calculate the volume of infarction. The size of the infarct volume was represented by the percentage of infarct volume to total brain volume.

### Immunofluorescence and immunohistochemical staining

Two hours after the end of the last salidroside administration at 3 or 7 days following the reperfusion, the animals were deeply anesthetized with isoflurane and perfused with 0.9% physiological saline followed by 4% paraformaldehyde solution from the left ventricle until the limbs were stiff. The brains were removed and postfixed in 4% paraformaldehyde and dehydrate in sucrose gradient for at least 48 h. Then, 30 μm thick frozen coronal sections were sliced using a freezing microtome and placed in antifreeze buffer and preserved in -20℃ before staining.

Immunofluorescence and immunohistochemical staining were performed using the methods in our previous research [29]. The sections containing the cortex, SVZ, striatum (bregma − 2.0 to − 1.0 mm; Paxinos and Watson, 2005) were selected. The immunofluorescence staining was performed on floating sections. For 5-Bromo-2’-Deoxyuridine (BrdU) staining, the sections were incubated in 2× SSC solution containing 10% formamide for 2 h at 65 °C. After rinsing in 2× SSC solution, the sections were incubated in 2 N hydrochloric acid for 30 min at 37 °C and rinsed in 0.1 M boric acid solution (pH 8.5) twice. After washing in 0.01 M phosphate-buffered saline (PBS), the sections were incubated in blocking reagent (0.01 M PBS with 5% bovine serum albumin and 0.5% Triton X-100) for 1 h at room temperature to block nonspecific binding. After that, the sections were double or triple labeled with an anti-BrdU antibody and another antibody of specific cell markers. The primary antibodies and the secondary antibodies used were showed in Table 1. The brain sections were incubated in the diluted antibodies at 4 °C overnight. Afterwards, the sections were washed and incubated in secondary antibodies for 1 h at 37 °C in a humidified chamber. The slides were observed on a confocal laser scanning microscope (Zeiss LSM710, Oberkochen, Germany).

For quantification, 2 discontinuous sections of each rat were selected, and 5 non-overlapping visual fields of each section were captured for the measurement. For the captured photo, the immunofluorescence intensity of BrdU and DCX was represented with the integrated optical density (IOD) of the whole photo, which was analyzed using Image-J 1.51 K software (National Institutes of Health, Bethesda, MD, USA). The immunofluorescence intensity of one animal was calculated from the average IOD of all the acquired photos of the animal. The quantification of the BrdU+/Nestin^+^/DCX^+^ or the BrdU^+^/Nestin^+^/GFAP^+^ cell numbers were carried out by observers who were blinded to the experimental grouping. The cell numbers of one animal were calculated from the sum of all the captured photos of the animal.

For immunohistochemical staining, the sections were incubated in 3% hydrogen peroxidase for 10 min to inhibit endogenous peroxidase activity. After that, sections were labeled with NeuN antibody at 4 °C overnight. Then sections were incubated with HRP-polymer anti-rabbit IgG working solution (Maixin Biotech. Co., Ltd., Fuzhou, China) for 15 min, and stained with diaminobenzidine (DAB) for 5 min. After that, sections were cleared with xylene, mounted on slides with neutral gum. The slides were observed on an inverted microscope (LEICA DMi8, Buffalo Grove, IL, USA). For quantification, the 2 discontinuous sections of each rat were selected, and 5 non-overlapping visual fields of each section were captured for the measurement. The NeuN^+^ cell numbers were counted using Image-J 1.51 K software.

**Table 1:** Antibodies used for immunofluorescence staining

Antibodies	Antigen	Host	Company	Catalog No.	Dilution
Primary antibody					
BrdU	Proliferating cell	Rat	Abcam	ab6326	1:200
DCX	Neuroblast	Rabbit	Abcam	Ab18723	1:1000
NeuN	Neuron	Rabbit	Abcam	Ab177487	1:1000
GFAP	Astrocyte	Rabbit	Abcam	Ab7260	1:1000
Nestin	Neural stem cell	Mouse	Abcam	Ab6142	1:200
Secondary Antibody(conjugation)					
Goat anti rabbit IgG (Alexa Fluor 488)	Rabbit IgG	Goat	Beyotime	A0423	1:200
Goat anti mouse IgG (Alexa Fluor 405)	Mouse IgG	Goat	Abcam	Ab175660	1:200
Goat anti rat IgG (cy3)	Rat IgG	Goat	Beyotime	A0507	1:200

### Enzyme-linked immunosorbent assays (ELISAs)

To determine the effects of salidroside on the brain tissue neurotrophins, the rats were sacrificed 7 days after the cerebral I/R. The brain tissue in peri-infarct area was dissected on ice and was homogenized (10% w/v) in saline. The protein concentration was essayed using a bicinchoninic acid Protein Assay Kit (Epizyme Biomedical Technology Co., Shanghai, China). The concentrations of brain-derived neurotrophic factor (BDNF) and nerve growth factor (NGF) were measured using the ELISA kit (F15100 and F16310, Westang Biotech Co. Ltd., Shanghai, China) according to the manufacturer’s instructions. The absorbance of wells was measured at 450 nm using a microplate reader (Thermo Varioskan Flash, Vantaa, Finland).

### Western blot assay

Proteins from the brain tissue of SVZ region was extracted using RIPA lysis Buffer (Beyotime Biotechnology Inc., Shanghai, China) according to the manufacturer’s instructions. The concentration of the protein was essayed using a bicinchoninic acid Protein Assay Kit (Epizyme Biomedical Technology Co., Shanghai, China). Then equal amounts of protein (50 µg) were loaded on a 10% SDS-PAGE for electrophoresis and then transferred to PVDF membranes. After blocking for 1 h in 5% non-fat milk powder dissolved in TBST buffer at room temperature, the membranes were incubated with the primary antibodies overnight at 4℃ (polyclonal rabbit anti-activated Notch1 antibody, 1:1000, Abcam, ab52301; polyclonal rabbit anti Hes-1 antibody, 1:1000, Abcam, ab71559;α-Tubulin Rabbit Polyclonal Antibody, 1:2000, Beyotime, AF5012). Then the membranes were incubated HRP-labeled goat anti-rabbit IgG (1:2000, Beyotime, A0208) at room temperature for 1 h. At last, the protein bands were visualized using an enhanced chemiluminescence kit (Beyotime Biotechnology Inc., Shanghai, China). Images were captured with a FluorChem M System (ProteinSimple, Santa Clara, CA, USA). The relative density of the bands was analyzed using Image-J 1.51 K software.

### Real-time reverse transcription-quantitative polymerase chain reaction (RT-qPCR) analyses

Total RNA was extracted from the brain tissue of peri-infarct region using Ultrapure RNA Kit (CWbio.Co.Ltd, Beijing, China) according to the manufacturer’s protocol. The concentration and purity of total RNA was measure in NanoDrop 2000 (Thermo, MA, USA). Complementary DNA (cDNA) was generated using HiFi-MMLV cDNA Kit (CWbio.Co.Ltd, Beijing, China). Dilute the excessively concentrated RNA to an appropriate ratio, resulting in a final concentration of 100–500 ng/µl. Quantitative RT-PCR was carried out on a ABI 7500 realtime fluorescence quantitative PCR apparatus (Applied Biosystems, CA, USA) using a UltraSYBR Mixture (With ROX) kit (CWbio.Co.Ltd, Beijing, China). Amplification procedure was set as follows: 95 °C 10 min for predenaturation, (95 °C 15 s→60 °C 60 s) ×45 circle, and 65℃→95℃ (the temperature rises by 0.3℃ every 15s) for dissolution curve determination. The oligonucleotide primer sequences were designed by NCBI BLAST as follows: BDNF (152 bp), Forward: CTACGAGACCAAGTGTAATC, Reverse: TTATGAATCGCCAGCCAAT; NGF (226 bp), Forward: GAGCGCATCGCTCTCCTT, Reverse: GAGCGCATCGCTCTCCTT; Notch1 (267 bp), Forward: CGCCCGTGGATTCATCTGTA, Reverse: GGGCATAGACAGCGGTAGAAAG; Hes1 (143 bp), Forward: CAAGCTGGAGAAGGCAGACAT, Reverse: CCTCGTTCATGCACTCGCTG; GADPH (138 bp), Forward: TGGAGTCTACTGGCGTCTT, Reverse: TGTCATATTTCTCGTGGTTCA. All samples were assayed triplicated, and relative gene expression was quantified using the 2^−ΔΔCT^ method using GADPH as a housekeeping gene.

### Statistical analysis

Data are presented as the mean ± SEM. All analyses were performed using the SPSS 20.0 software (SPSS Inc., Chicago, IL, USA). The multiple group comparisons were assessed by one-way analysis of variance, followed by LSD post hot test when the data was normal distributed and conformed to homogeneity of variance. And Games-Howell post hot test was employed when the data was normal distributed and did not conform to homogeneity of variance. *p* values less than 0.05 were considered statistically significant.

## Results

### Salidroside improved neurological function recovery after I/R

**Fig. 2:**
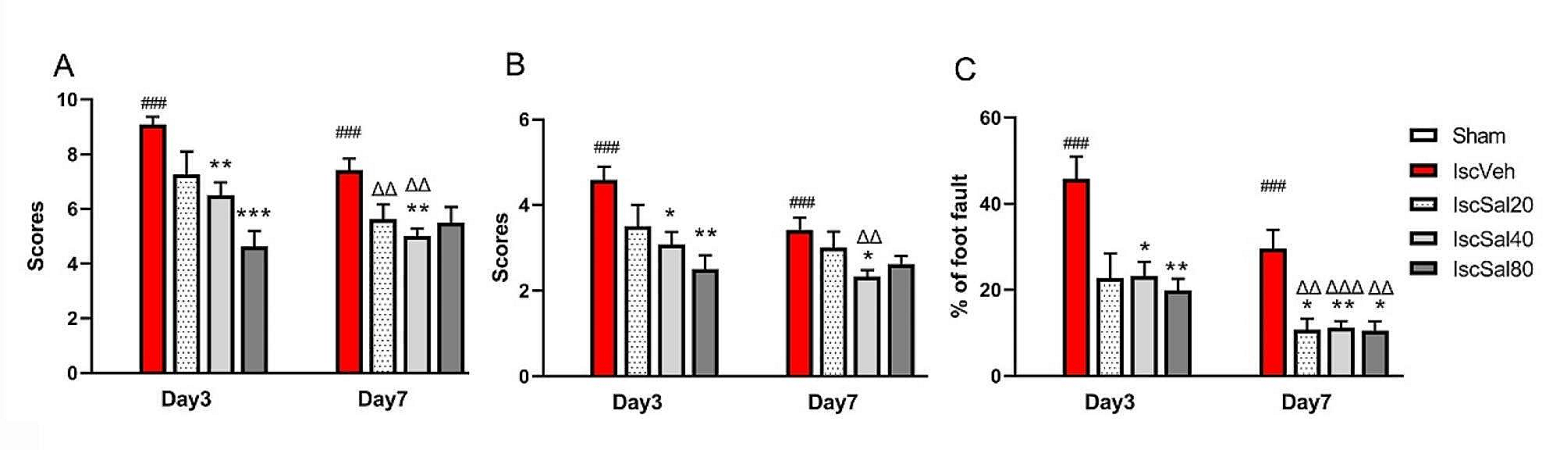
Salidroside improved neurological function recovery by decreased the mNSS scores (**A**), the beam balance scores (**B**), and percent of foot fault (**C**) after MCAO. Rats were treated 20, 40 or 80 mg/kg salidroside, or saline water per day, by intraperitoneal injection. Data are expressed as mean ± SEM (*n* = 12 in the sham, IscVeh and IscSal40 groups, *n* = 8 in the IscSal20 and IscSal80 groups). Multiple group comparisons were assessed by one-way analysis of variance, followed by LSD-*t* test. ^###^*p* < 0.001 compared to the sham group. *p#x2009;< 0.05, **p < 0.01, ***p#x2009;< 0.001, compared to the IscVeh group. ^Δ^*p* < 0.05, ^ΔΔ^*p* < 0.01, ^ΔΔΔ^*p* < 0.001, compared to the corresponding scores at time point Day 3

Modified neurological severity scoring (mNSS), beam balance test, and foot fault test were performed to evaluate the effects of salidroside on cerebral ischemia induced neurobehavioral impairments (Fig. 2). Rats in the sham group did not show any functional deficit, while in IscVeh group, the mNSS scores, beam balance scores, as well as the foot fault percentage had severely increased (Fig. 2A, B and C, *p* < 0.001) compared to those of the sham group. Salidroside treatment dose-dependently decreased the mNSS scores, the beam balance scores and the percentage of foot fault significantly (Fig. 2A, B and C, *p* < 0.05 or *p* < 0.01 at different time points) compared to those of the IscVeh group. Moreover, 7 days of salidroside treatment led to better behavioral recovery than 3 days of treatment (*p* < 0.01 or *p* < 0.05 in different behavioral tests). The data showed that salidroside may ameliorate the neurological function loss after cerebral I/R.

### Salidroside reduced the volume of cerebral infarction

**Fig. 3:**
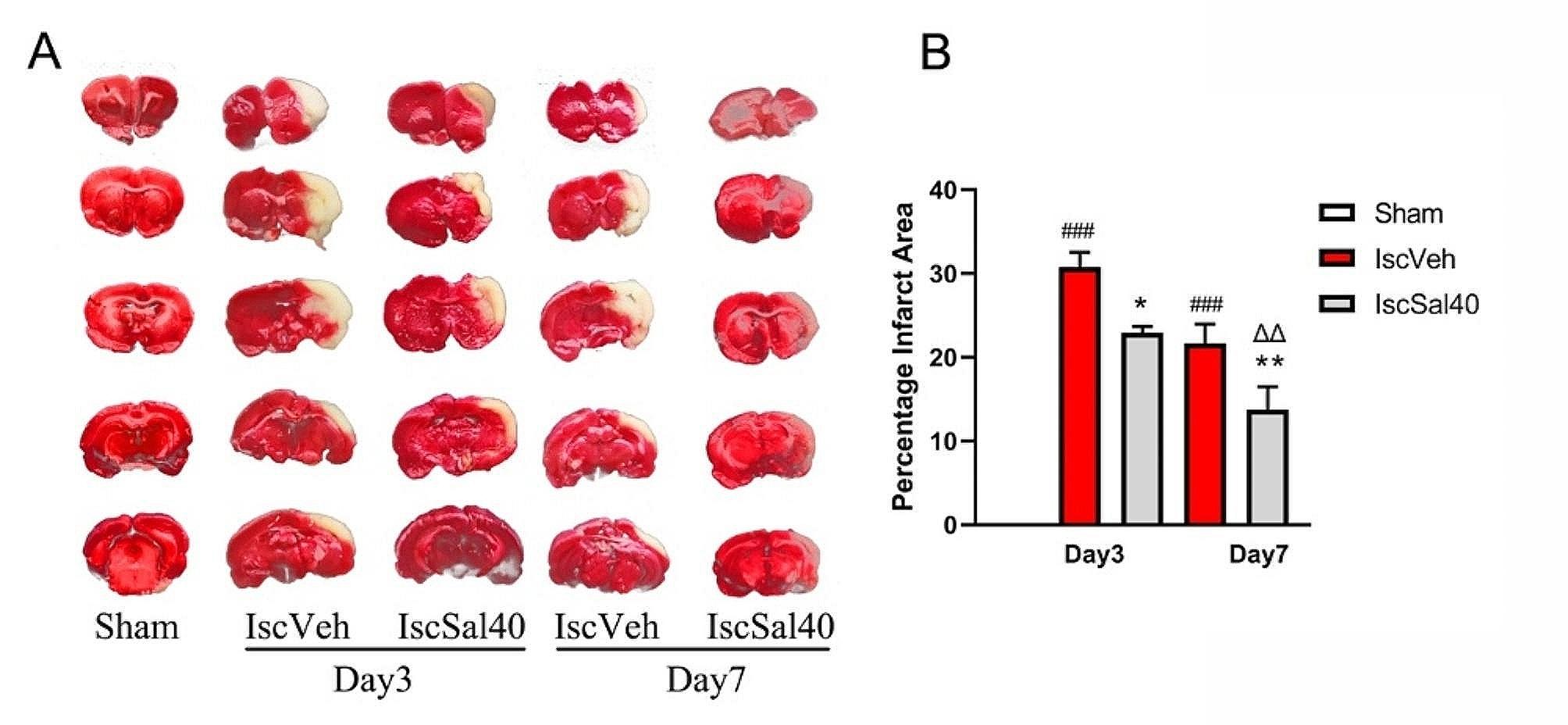
Salidroside reduced the volume of cerebral infarction. (**A**), Representative images of brain tissue TTC staining. White colour represented ischemic area. (**B**) Infarct percentage calculated from 5 animals in each group. Data are expressed as mean ± SEM. ^###^*p* < 0.01, compared to the sham group. *p#x2009;< 0.05, **p < 0.01, compared to the IscVeh group. ^ΔΔ^*p* < 0.01, compared to the IscSal40 group at time point Day 3

TTC staining results showed that MCAO surgery caused severe cerebral ischemia, and treatment with salidroside significantly reduced cerebral infarct volume after cerebral ischemia (Fig. 3). 7 days of administration led to a greater reduction in cerebral infarction volume (*p* < 0.01).

### Salidroside elevated the survival of neurons

**Fig. 4:**
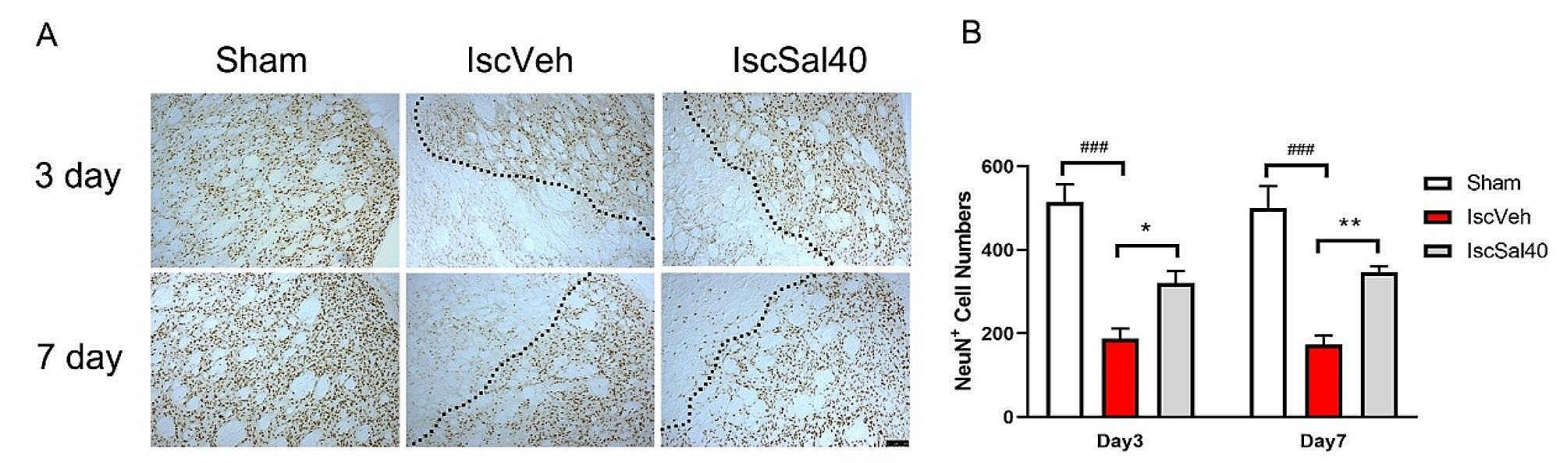
Salidroside increased the numbers of NeuN + cells, and the immunoreactivity area of NeuN expression in ischemic striatum after MCAO. (**A**) NeuN IHC staining in striatum, 3 days or 7 days after MCAO. The dotted line showed the barrier of NeuN + areas. Scare bar, 100 μm. (**B**) NeuN + cell numbers in striatum. Data are expressed as mean ± SEM (*n* = 5 in the sham, IscVeh, IscSal40 groups). Multiple group comparisons were assessed by one-way analysis of variance, followed by LSD-*t* test. ^###^*p* < 0.001 compared to the sham group. *p < 0.05, **p < 0.01, compared to the IscVeh group

NeuN is a neuronal specific nuclear protein which expression can be observed in most types of neuron cells throughout the nervous system. Thus, we use NeuN as a biomarker to investigate the overall survival of neuron cells (Fig. 4). After MCAO surgery, the NeuN^+^ cell numbers in ischemic striatum decreased significantly, and the immunoreactivity area of NeuN expression shrunk (Fig. 4A, B, *p* < 0.001) compared to the sham group. The NeuN^+^ cell numbers substantially raised after salidroside treatment. Furthermore, the immunoreactivity area of NeuN expression enlarged (*p* < 0.05 or *p* < 0.01 at different time points) compared to the IscVeh group. These results suggest that salidroside may promote the survival of neuron cells after cerebral I/R.

### Salidroside promoted neural regeneration after I/R

**Fig. 5:**
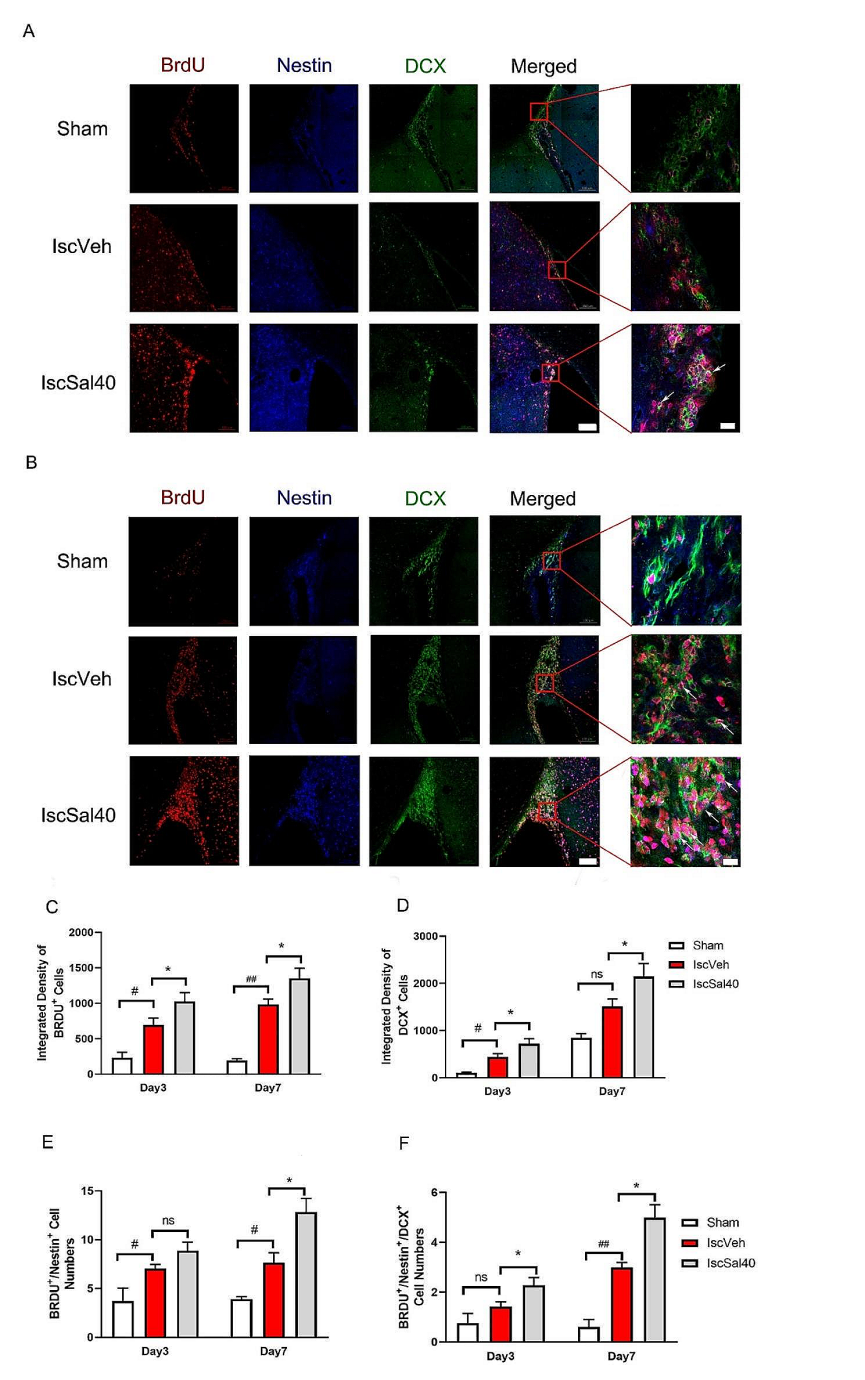
Effect of salidroside on BrdU^+^/Nestin^+^/DCX^+^ immunoreactivity in ischemic hemisphere. Red fluorescence from Cy3 indicates BrdU, blue fluorescence from FITC indicates Nestin, and green fluorescence from Alexa Fluor 488 indicates DCX. The arrows show the co-localization of BrdU^+^/Nestin^+^/DCX^+^. Scale bars: 100 μm and 50 μm for enlarged images. (**A**) 3 days after MCAO. (**B**) 7 days after MCAO. (**C**) Quantification of immunofluorescence intensity of BrdU^+^ cells in the ischemic hemisphere. (**D**) Quantification of immunofluorescence intensity of DCX^+^ cells in the SVZ. (**E**) Numbers of BrdU^+^/Nestin^+^ cells in SVZ. (**F**) Numbers of BrdU^+^/Nestin^+^/ DCX^+^ cells in SVZ. Data are expressed as mean ± SEM (*n* = 3 in the sham, *n* = 5 in IscVeh, IscSal40 groups). Multiple group comparisons were assessed by one-way analysis of variance, followed by LSD-*t* test. ^#^*p* < 0.05, ^##^*p* < 0.01, compared to the sham group. *p < 0.05, **p < 0.01, compared to the IscVeh group. ns: no significance

**Fig. 6:**
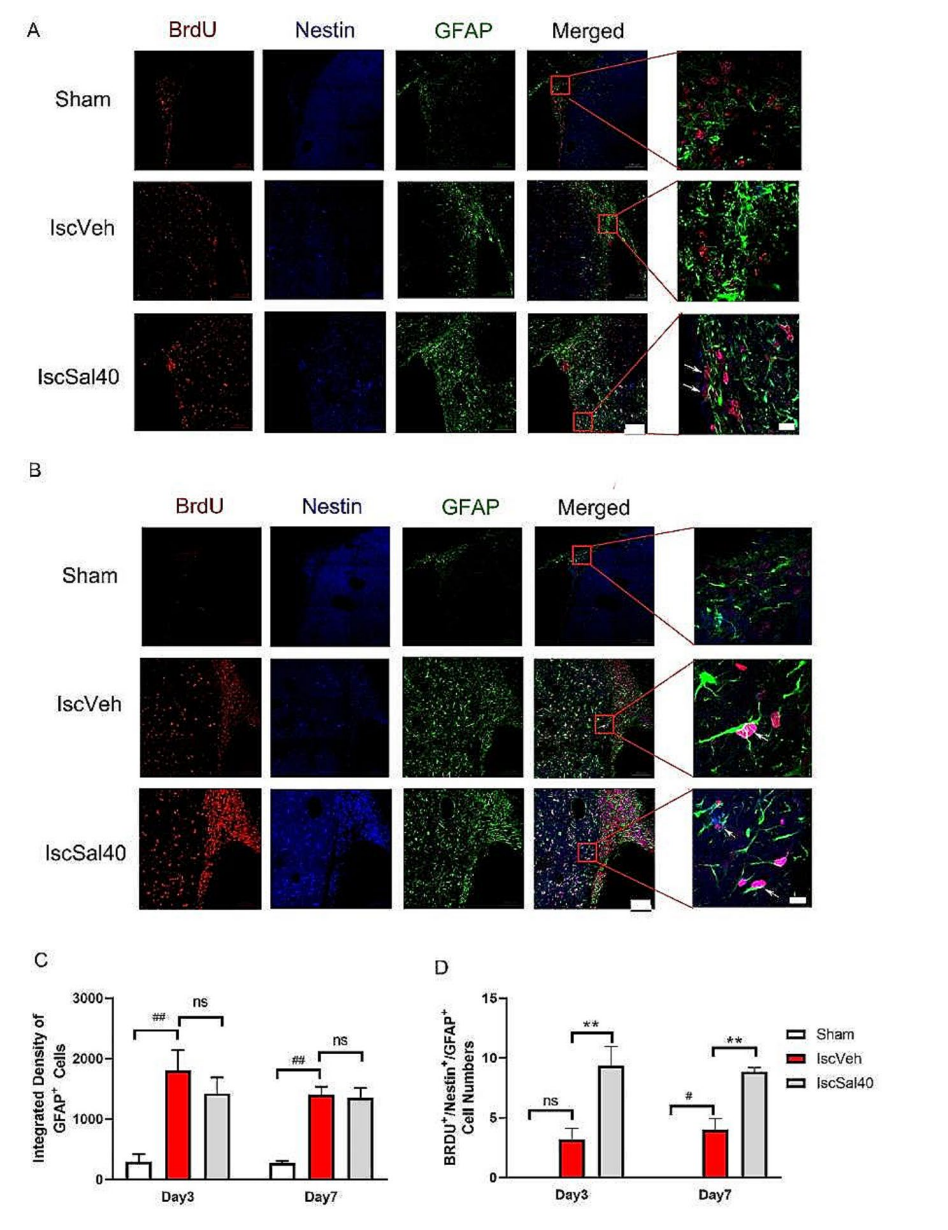
Effect of salidroside on BrdU^+^/Nestin^+^/GFAP^+^ immunoreactivity in ischemic hemisphere. Red fluorescence from Cy3 indicates BrdU, blue fluorescence indicates Nestin, and green fluorescence from Alexa Fluor 488 indicates GFAP. The arrows show the co-localization of BrdU^+^/Nestin^+^/GFAP^+^. Scale bars: 100 μm and 50 μm for enlarged images. (**A**) 3 days after MCAO. (**B**) 7 days after MCAO. (**C**) Quantification of immunofluorescence intensity of GFAP^+^ cells in striatum. (**D**) Quantification of number of BrdU^+^/Nestin^+^/ GFAP^+^ cells in striatum. Data are expressed as mean ± SEM (*n* = 3 in the sham, *n* = 5 in IscVeh, IscSal40 groups). Multiple group comparisons were assessed by one-way analysis of variance, followed by LSD-*t* test. ^#^*p* < 0.05, ^##^*p* < 0.01 compared to the sham group. **p < 0.01 compared to the IscVeh group. ns: no significance

Some biomarkers can be used to display the situation of neural regeneration and differentiation. In particular, 5-Bromo-2’-Deoxyuridin (BrdU) can identify proliferating cells, Nestin is known as a neural stem/progenitor cell marker, while doublecortin (DCX) is the biomarker of neural precursor cells, and glial fibrillary acidic protein (GFAP) is astrocytes biomarker. We investigated the expression and location of labeled cells, especially double/triple labeled co-localized cells, in ischemic striatum and adjacent subventricular zone (SVZ) (Figs. 5 and 6).

SVZ is one of the “stem cell niche” in brain. In the sham group, small amount of BrdU^+^ cells existed in SVZ. After I/R, some BrdU^+^ cells emerged in striatum. The intensity of BrdU immunoreactivity increased in both striatum and SVZ 3 days after MCAO surgery (Fig. 5A, B, C) compared to the sham group, which meant the proliferation of neural cells enhanced after cerebral I/R. Some Nestin positive neuroepithelial cells were also proliferating after cerebral I/R. In the IscVeh group, BrdU^+^/Nestin^+^ cell numbers significant increased (Fig. 5A, B, E, *p* < 0.01) compared to the sham group.

DCX positive neural precursor cells, or so called immature neurons, distributed only in SVZ. These cells have ability to develop into the regeneration neurons cells. The integrated density of them increased slightly at 7 days after MCAO (Fig. 5A, B, D), compared to the sham group. Meanwhile, BrdU^+^/Nestin^+^/DCX^+^ cell numbers in SVZ augmented at 7 days after MCAO (Fig. 5A, B, F, *p* < 0.01) compared to the sham group.

After salidroside administration, the intensity of BrdU^+^ augmented significantly in 7 days after MCAO (Fig. 5A, B, C, *p* < 0.05) compared to the IscVeh group. Similarly, the DCX immunoreactivity increased at 7 days after salidroside treatment (Fig. 5A, B, D, *p* < 0.01), compared to the IscVeh group. Salidroside treatment enhanced BrdU^+^/Nestin^+^ cells numbers in both SVZ and striatum (Fig. 5A, B, E, F, *p* < 0.01), and also BrdU^+^/Nestin^+^/DCX^+^ cell numbers in SVZ (Fig. 5A, B, F, *p* < 0.01) compared to the IscVeh group, at 7 days following reperfusion. In conclusion, salidroside treatment may augment neural regeneration through enhancing the neural precursor cells and stem cells proliferation.

Astrocytes activated after brain injury, as the result, the intensity of GFAP immunoreactivity enhanced significantly after MCAO (Fig. 6A, B, C, *p* < 0.01). After cerebral I/R, some GFAP^+^ cells are also labeled by BrdU and Nestin, which could mainly be observed in both striatum and SVZ. Salidroside treatment did not influence the GFAP expression (Fig. 6A, B, C), but it could further augment the BrdU^+^/Nestin^+^/GFAP^+^ cell numbers at 3 days after I/R (Fig. 6A, B, D, *p* < 0.01) compared to the IscVeh group. In summary, though salidroside did not influence the up-regulation of GFAP after ischemic injury, it may increase the number of proliferated reactive astrocytes which expressed the proliferation marker BrdU and the stem cell hallmarkers like Nestin.

### Effect of salidroside on cerebral BDNF and NGF protein levels and mRNA expression

**Fig. 7:**
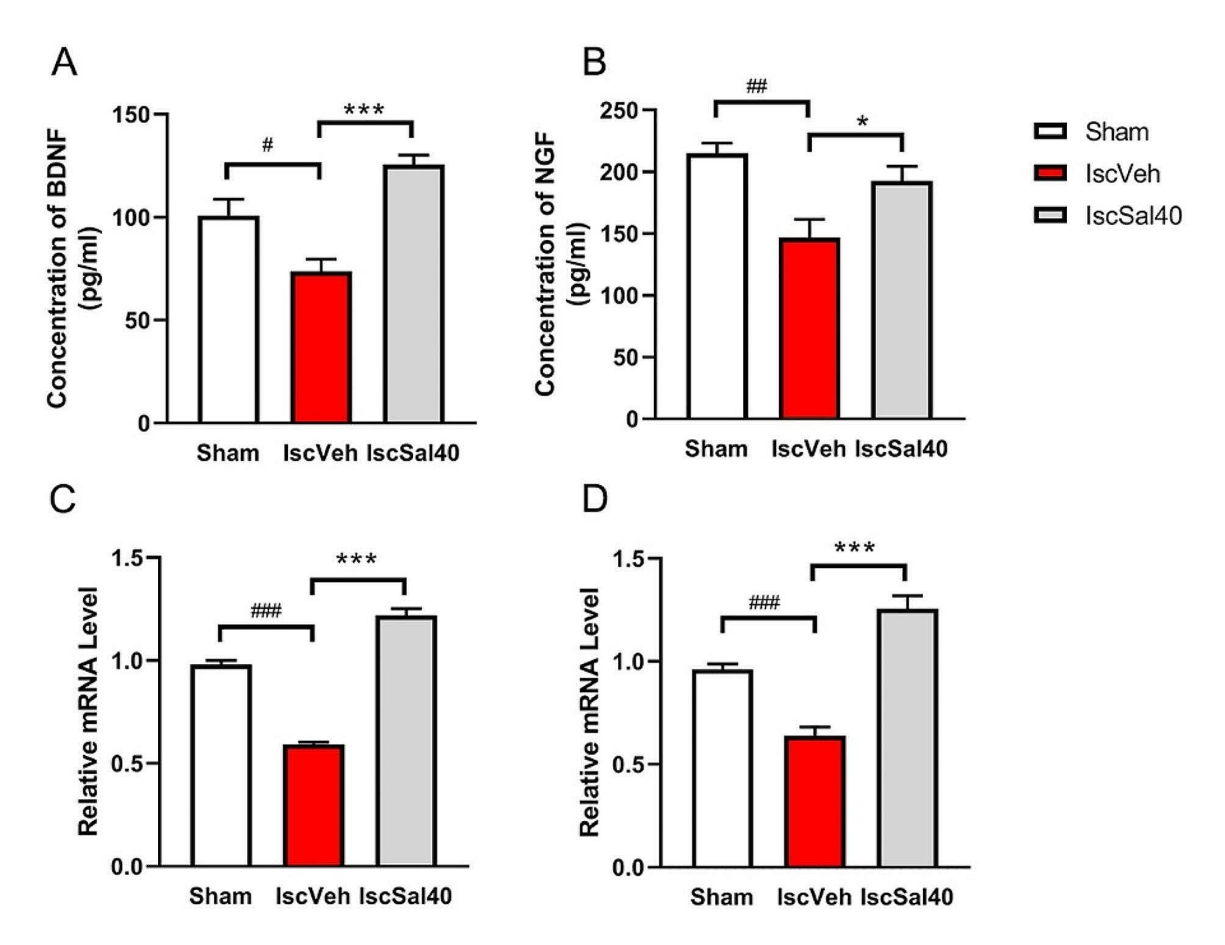
Salidroside increased the protein level and mRNA expression of BDNF and NGF at 7 days after MCAO Concentration of BDNF (**A**) and NGF (**B**) in peri-infarct area were determined by ELISA. Relative mRNA levels of BDNF (**C**) and NGF (**D**) in ischemic area were determined by RT-PCR. Data are expressed as mean ± SEM (*n* = 7 in each group). Multiple group comparisons were assessed by one-way analysis of variance, followed by LSD-*t* test. ^#^*p* < 0.05, ^##^*p* < 0.01, ^###^*p* < 0.001 compared to the sham group. *p < 0.05, ***p < 0.001, compared to the IscVeh group

BDNF and NGF are common neurotrophins which regulate development, maintenance and function of nervous systems. The mRNA expression of BDNF and NGF were determined. Relative mRNA levels of both neurotrophins dropped sharply after MCAO surgery (Fig. 7C, D, *p* < 0.001) compared to the sham group. After salidroside treatment, the mRNA levels of BDNF and NGF significantly raised (Fig. 7C, D, *p* < 0.001) compared to the IscVeh group. The protein levels of neurotrophins were quantitative by ELISA. Both BDNF and NGF protein levels decreased after MCAO (Fig. 7A, B, *p* < 0.05) compared to those of the sham group. Salidroside treatment elevated BDNF and NGF level significantly (Fig. 7A, B, *p* < 0.05) compared to the IscVeh group. Based on these results, combined with the protein level change of BDNF and NGF, salidroside may contribute to the neurotrophins secretion after cerebral I/R.

### Effect of salidroside on the mRNA and protein expression of Notch and Hes1

**Fig. 8:**
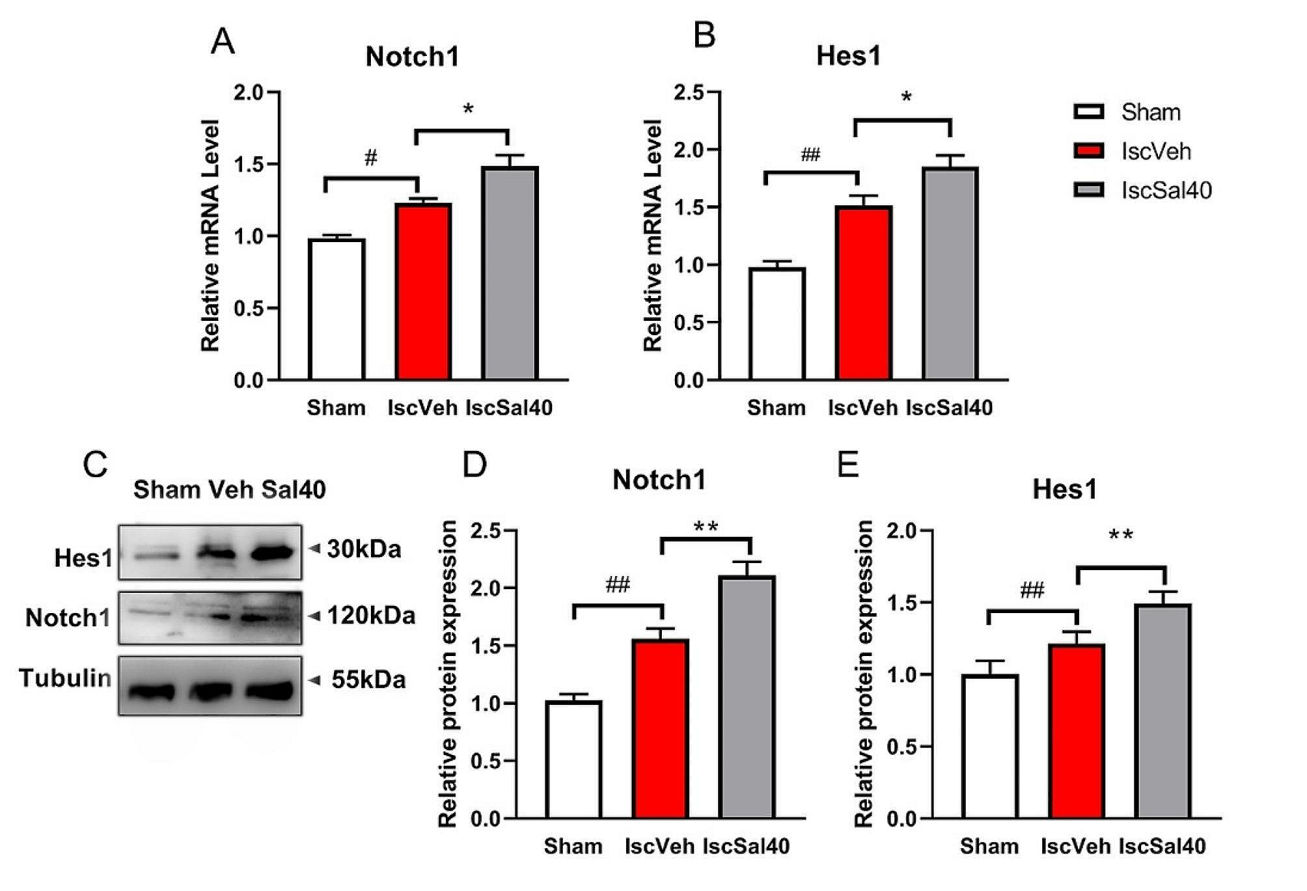
Salidroside promoted the Notch1 and Hes1 mRNA and protein expression after MCAO. Relative mRNA levels of Notch1 (**A**) and Hes1 (**B**) in ischemic SVZ were determined by RT-PCR. Results are presented as mean ± SEM (*n* = 5 in the sham, *n* = 7 in the IscVeh and IscSal40 group). (**C**) Representative images of western blot assay for Notch1 and Hes1. Quantification of integrated density for Notch1 (**D**) and Hes1 (**E**), normalized to Tubulin. Results are presented as mean ± SD (*n* = 3 in the sham, *n* = 5 in the IscVeh and IscSal40 group). Multiple group comparisons were assessed by one-way analysis of variance, followed by LSD-*t* test. ^#^*p* < 0.05, ^##^*p* < 0.01, compared to the sham group. *p < 0.05, **p < 0.01, compared to the IscVeh group

In order to explore the mechanism by which salidroside promoted the proliferation and differentiation of endogenous neural stem cells, we analyzed the protein level and mRNA expression of Notch signaling pathway and its target molecular, Hes1, in ischemic SVZ at 7 days after MCAO (Fig. 8). In IscVeh group, the mRNA and protein level of Notch1 enhanced compared to the sham group. After salidroside administration, the expression of Notch1 was significantly promoted compared to the IscVeh group (Fig. 8A, C and D, *p* < 0.01). The mRNA and protein level of Hes1 increased compared to the sham group, and salidroside treatment elevated Hes1 expression, compared to the IscVeh group (Fig. 8B, C and E, *p* < 0.05). Generally speaking, salidroside may activate the Notch pathway after cerebral I/R.

## Discussion

In the present study, we investigated the neuroprotective effects of salidroside on cerebral I/R and its activity on neural regeneration. Salidroside treatment significantly improved the neural behavior performance. Immunohistochemistry and multiple-labeled fluorescence staining analysis showed that salidroside promoted the proliferation of neuroblast (indicated by BrdU^+^/Nestin^+^/DCX^+^ triple-staining) in SVZ and reactive astrocyte (indicated by BrdU^+^/ Nestin^+^/GFAP^+^ triple-staining) in peri-ischemic area. Furthermore, salidroside elevated the mRNA and protein levels of BDNF and NGF, as well as the mRNA and protein level of Notch1/Hes1, which may involve in the mechanism of the neurogenesis promotional effects of salidroside.

Effects of salidroside on cerebral ischemic injury are well documented before. Previous studies have reported that salidroside exerts neuroprotective effects on cerebral I/R rats at the dosage of 20–100 mg/kg in a dose-dependent-manner [15, 17, 32–34]. Our current findings showed that the neurobehavioral impairment caused by cerebral ischemia-reperfusion injury has a certain dose-dependency in the treatment with salidroside at dosage of 40 mg/kg and 80 mg/kg. This is consistent with reports from others. Since a dosage of 40 mg/kg of salidroside already exhibits a significant therapeutic effect, we employed this dosage to evaluate the effect of salidroside on promoting neuroregeneration following cerebral ischemia in the subsequent experiments.

DCX, a microtubule-associated protein expressed in newborn neurons [35], is usually used as marker of neural precursor cells. Nestin, an intermediate filament protein in the neuroepithelial stem cell and radial glia, is the marker of embryonic NSCs, whose expression persists until astrocyte development. The present study found that many BrdU^+^ proliferating cells appeared in the SVZ region after ischemic injury. A great number of them coexisted with DCX. At the same time, BrdU^+^ cells were also labeled by Nestin incorporation in the injured area. BrdU^+^/Nestin^+^ and BrdU^+^/DCX^+^ cells are located in different but overlapping regions, indicating that cerebral ischemia stimulated obvious proliferation of endogenous neuroblasts. This phenomenon is consistent with many other literature reports [36]. Salidroside was found to increase numbers of BrdU^+^/Nestin^+^/DCX^+^ cells in SVZ region, suggesting that salidroside may promote neuroblast proliferation after cerebral ischemia.

Astrocytes, as the most numerous glial cell type in the CNS, play a critical role in regulating homeostasis, increasing synaptic plasticity and maintenance of brain architecture as well as function. They react to cerebral ischemic injury by hypertrophy and up-regulation of GFAP, the common phenomenon termed as reactive astrogliosis. Moreover, reactive astrocytes are not only morphologically changed, but some of them also proliferated. Approximately 15–40% of all astrocytes become proliferating astrocytes, labelled by GFAP and BrdU double staining [37]. As reported in our present study, in the peri-injury region of the striatum, most of the proliferating astrocytes were labelled by Nestin, suggesting that reactive astrocytes regained the progenitor marker. This phenomenon was also previously observed by other researchers [38]. Behavioral studies showed that reactive astrocyte proliferation and astrogliosis correlate with neuronal remodeling and functional recovery [39]. Reactive astrocytes exert beneficial effects on brain ischemic injury by limiting damage locally through the production of glial scars [40]. Using some reagent to inhibit astrocytes or knocking out GFAP genes impairs or delays neurological recovery [41]. Proliferating reactive astrocytes may increase neuroplasticity and improve neurovascular remodeling, thus contributing to stroke outcomes [42]. The presence of GFAP/Nestin^+^ cells suggests that cerebral ischemia induces developmental phenotypes with differentiation potential and changes the direction of differentiation within neuroglia cell populations [37]. In the present study, although no influence of salidroside on the expression of GFAP was found, treatment of salidroside significantly increased the proliferation of reactive astrocytes credibly, and these cells expressed progenitor markers, suggesting that salidroside may have potential effects to redirect astrocyte differentiation induced by ischemia, and this effect may have implication for neuroplasticity. Indeed, salidroside has been reported to promote dendritic and synaptic plasticity [32].

BDNF and NGF are both endogenous proteins involved in neuronal development, differentiation and survival. BDNF plays an important role in promoting neural regeneration, regulating synaptic plasticity and functional recovery from cerebral ischemia [43]. Many studies reported decreased BDNF level after stroke [44], and treatments or drugs that boost BDNF level have been proven to contribute to rehabilitation after stroke [45, 46]. NGF plays an important role including regulation of the growth, development and plasticity of neural populations in the nervous system. NGF elevates the survival of neural cells in vivo and in vitro. It has the ability to protect against delayed neuronal death after cerebral ischemia [47]. Similar to BDNF, NGF expression is downregulated after cerebral I/R, and exogenous NGF administration in the early post-ischemic period attenuate brain-infarct and improve neurobehavioral assessment [48]. NGF can induce migration of the astrocytes, but does not affect proliferation [49]. The present study found that salidroside promoted the transcription of BDNF and NGF. Moreover, the protein level of them were also elevated by salidroside treatment. Research shows that protective effect of salidroside against cerebral ischemic injury is inhibited in BDNF knockout mice [50]. And salidroside affects Schwann cells growth through the modulation of neurotrophic factors (BDNF, GDNF and CNTF) [51]. These studies and our present findings suggest that upregulation of neurotrophic factors such as BDNF and NGF may be an important mechanism by which salidroside exerts multiple neuroprotective effects.

Activation of the Notch signaling pathway has neuroprotective effects against cerebral ischemia [52]. Molecules of Notch signaling pathway expressed after cerebral I/R. Notch1 was predominantly expressed in DCX positive cells, whereas both Notch1 and its ligand Jagged1, expressed in astrocytes but not microglia in the peri-infarct region, and the activated form of Notch1, as well as Notch1 downstream transcriptional target Hes1 mainly expressed in SVZ [28]. In our study, we found that salidroside treatment enhanced Notch1 and Hes1 expression in SVZ at 7 days after MCAO, suggesting that salidroside may activate Notch signaling, which relates to the proliferation of NSCs. So far, a few researches reported the involvement of Notch signaling pathway in the regulation effects of salidroside in different pathological state. Salidroside induces neuronal differentiation of mouse mesenchymal stem cells through Notch signaling [53]. Salidroside suppresses the metastasis of hepatocellular carcinoma cells [54], and inhibit lipopolysaccharide-ethanol- induced activation of proinflammatory macrophages [55] via Notch signaling pathway. Our study reported the regulation of Notch signaling by salidroside in cerebral I/R, suggesting that Notch signaling pathway may be involved in the possible mechanism of salidroside to promote neural regeneration after cerebral I/R. In addition, recent study revealed that the elevation effect of rosuvastatin on BDNF expression could be partially suppressed by DAPT, the Notch 1 pathway inhibitor [56]. BDNF may be an indirect target of Notch1 [57]. These researches indicated some relationship between Notch pathway and BDNF expression.

Cerebrovascular ischemic injuries occur in a time-dependent manner and are categorized as acute (minutes to hours), subacute (hours to days), or chronic (days to months). Day 3 and day 7 after ischemia are considered to be the subacute phase of cerebral ischemic injury [58]. Reports showed that neural progenitor cells initiate proliferation as early as day 1 following the onset of ischemia [4]. Our observation is that treatment with salidroside can enhance the proliferation of neural progenitor cells at subacute stage (day 3 and day 7) after cerebral ischemia. The results indicate that salidroside has a positive role in promoting the proliferation of neural progenitor cells and promotes the acquisition of stemness in astrocytes. The results suggested that salidroside may have a repairing effect on cerebral ischemic damage from the early stages of treatment.

Although current research provides evidence that salidroside promotes neural regeneration after cerebral ischemia, there are still many gaps that need to be filled. The time points (3 days and 7 days) we selected to observe the effects of salidroside were not long enough to observe its effects on the differentiation of these proliferating neural progenitor cells into neurons or glial cells. Differentiation requires a longer period of time (14 days or 28 days). So the long term effects of salidroside are the focus of our future research. Furthermore, more studies are needed to determine how salidroside influence the circuits between Notch signaling pathway and neural cells, whether BDNF and NGF involve in the underlying mechanism. Recently it was revealed that the elevation effect of rosuvastatin on BDNF expression could be partially suppressed by DAPT, the Notch 1 pathway inhibitor in an in vitro study in primary cortical neuron [56]. BDNF may be an indirect target of Notch1 in a research of Alzheimer’s disease [57]. These researches indicated some relationship between Notch pathway and BDNF expression. However, in our study, Notch pathway was found to be activated after cerebral ischemia, while BDNF was decreased, showing a different trend after ischemic injury. BDNF, as a major neurotrophic factor, was regulated by many upstream genes. The relationship between Notch pathway and BDNF after cerebral ischemia and the role of salidroside still need to be further explored.

## Conclusion

In summary, our current work demonstrates that salidroside promotes neurological function recovery after cerebral I/R, and enhances the proliferation of neural progenitors and reactive astrocytes. The mechanism may involve the effect of salidroside elevating BDNF/NGF expression and activating Notch signaling pathway. This study adds to the understanding of the mechanism of neural protective effect of salidroside.

### Electronic supplementary material

Below is the link to the electronic supplementary material.

Supplementary Material 1

Supplementary Material 2

## Data Availability

The data in the manuscript are from experiment results. More detailed supporting data can be provided upon request. Kan Lin could be contacted if someone wants to request the data from this study.
