# Supplementary material for: The effect of salidroside in promoting endogenous neural regeneration after cerebral ischemia/reperfusion involves notch signaling pathway and neurotrophic factors
Source: BMC Complement Med Ther. 2024 Aug 1;24:293. doi: 10.1186/s12906-024-04597-w (PMC11295647; doi:10.1186/s12906-024-04597-w)
Supplement: Supplementary file 1 — Supplementary Material 1 [file 12906_2024_4597_MOESM1_ESM.docx]

Supplemental Table 1. Mortality rate of all MCAO groups.

| **Experiments** | **Groups** | **Mortality rate** |
| --- | --- | --- |
| Behavior scoring  IHC/IF staining  RT-PCR  Elisa  Western-Blot | Sham （3day、7day） | 0 (0/17) |
|  | IscVeh（3day、7day） | 19.0% (4/21) |
|  | IscSal20（7day） | 20% (2/10) |
|  | IscSal40（3day、7day） | 10.5% (2/19) |
|  | IscSal80（7day） | 0 (0/8) |
| TTC staining | Sham | 0 (0/5) |
|  | IscVeh（3day、7day） | 16.7% (2/12) |
|  | IscSal40（3day、7day） | 16.7% (2/12) |
| **Total** | MCAO | 14.6%（12/82） |
|  | Sham | 0 (0/22) |
