# Supplementary material for: The effect of salidroside in promoting endogenous neural regeneration after cerebral ischemia/reperfusion involves notch signaling pathway and neurotrophic factors
Source: BMC Complement Med Ther. 2024 Aug 1;24:293. doi: 10.1186/s12906-024-04597-w (PMC11295647; doi:10.1186/s12906-024-04597-w)

Original full length western blots

The following figures showed the full length bolts of Hes-1 or Notch1. Images of Figure 8C were cropped from these blots. The membrane was exposed as a whole. The loading control (Tublin) was exposed at the first time, and the target band (Hes-1 or Notch1) was made for a second exposure by shielding the control band(Tubin) with a piece of black plate.

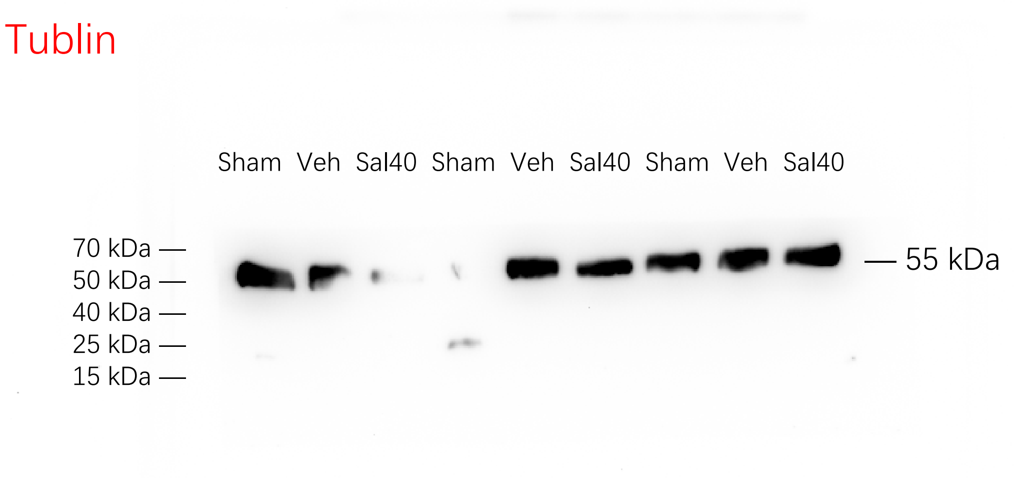

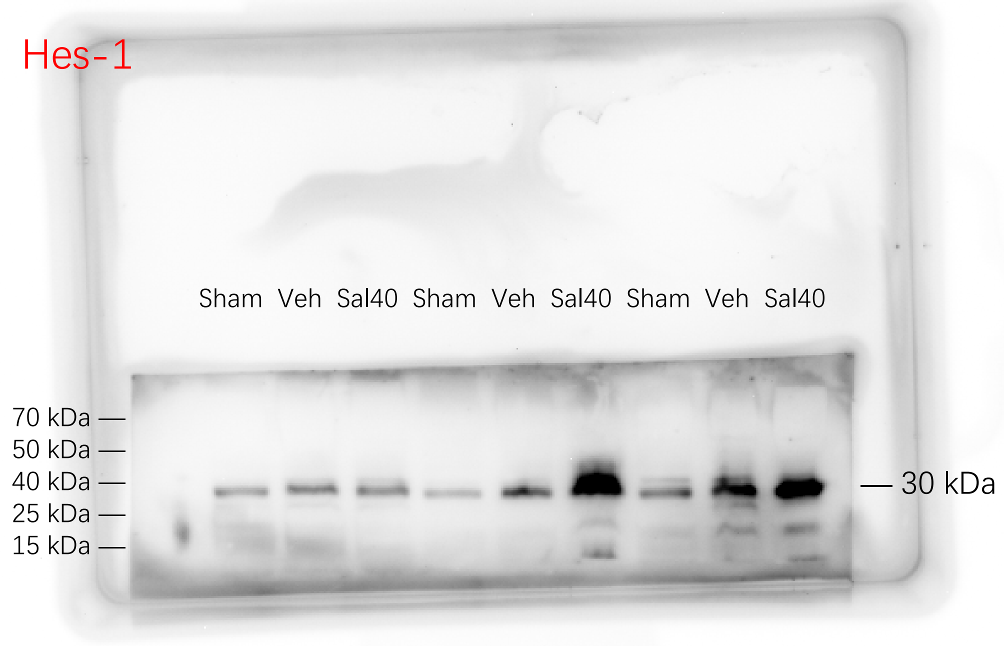

Tublin

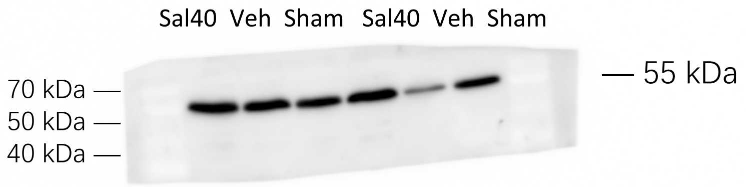

Notch1

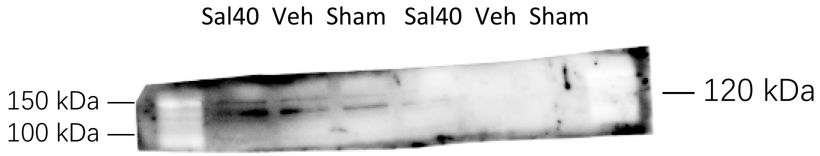

Supplement: Supplementary file 2 — Supplementary Material 2 [file 12906_2024_4597_MOESM2_ESM.pdf]
